# Ultra-High-Density Tripotassium 4,5-Bis(gem-dinitromethyl)-1,2,3-triazolate Hydrate (3K_3_BNOT·4H_2_O): A Lead-Free Triazole-Based Energetic Salt

**DOI:** 10.3390/molecules31121992

**Published:** 2026-06-07

**Authors:** Ruokai Pei, Yang Wu, Yinglei Wang

**Affiliations:** 1 Institute of Energetic Materials, Xi’an Modern Chemistry Research Institute, Xi’an 710065, China; p18955883901@163.com (R.P.); wuy_204@163.com (Y.W.); 2 State Key Laboratory of Fluorine & Nitrogen Chemicals, Xi’an 710065, China

**Keywords:** 1,2,3-triazole, energetic potassium salt, coordination network, gem-dinitro group, detonation performance

## Abstract

Energetic materials face dual challenges of enhancing detonation performance and replacing toxic lead-based formulations. Triazole-based energetic potassium salts typically struggle to achieve simultaneous high-density and excellent detonation properties. Herein, a novel gem-dinitro-functionalized 1,2,3-triazole energetic salt, tripotassium 4,5-bis(gem-dinitromethyl)-2H-1,2,3-triazolate (3K_3_BNOT·4H_2_O), was rationally designed and synthesized via a six-step mild route using diaminomaleonitrile as the starting material. The structure was fully characterized by IR, NMR, elemental analysis, and single-crystal X-ray diffraction (SC-XRD). 3K_3_BNOT·4H_2_O crystallizes in the triclinic system (space group P-1) and forms a three-dimensional K-O/K-N ionic coordination network, delivering an ultra-high anhydrous crystal density of 2.077 g·cm^−3^ at 193K. It exhibits a peak decomposition temperature of 183.8 °C (10 °C·min^−1^), impact sensitivity of 5 J, and friction sensitivity of 60 N (standard BAM methods). The calculated detonation velocity and pressure reach 8836 m·s^−1^ and 28.6 GPa, respectively, outperforming the classical explosive RDX. This work provides a structural analysis of triazole-based energetic potassium salt hydrates, and 3K_3_BNOT·4H_2_O shows structural potential as a high-energy energetic material; its initiating performance needs further experimental verification.

## 1. Introduction

Energetic materials are critical for both military and civilian pyrotechnic systems, yet the field currently confronts two urgent challenges: breaking the upper limit of energy output and achieving green replacement of lead-based primary explosives [1,2]. Conventional primary explosives such as lead styphnate (LTNR) and lead azide (LA) cause severe heavy metal pollution and occupational health hazards due to their high lead content [3]. Long-term exposure to lead fumes and residues leads to irreversible damage to the nervous and hematopoietic systems, driving the urgent development of high-performance lead-free alternatives [4].

Nitrogen-rich heterocyclic energetic compounds have emerged as promising candidates owing to their high heats of formation, high density, and environmentally benign detonation products dominated by N_2_ [5]. Among them, 1,2,3-triazole frameworks possess stable conjugated systems, high nitrogen content, and abundant reactive sites, making them ideal scaffolds for constructing energetic metal salts [6]. The gem-dinitro group, as a strong electron-withdrawing explosophoric group, can significantly improve oxygen balance and detonation performance [7]. However, most reported triazole-based energetic potassium salts have crystal densities below 2.0 g·cm^−3^, and only a few exceed 2.05 g·cm^−3^, limiting their detonation performance [8,9]. Additionally, the controlled synthesis and structure-property relationships of bis(gem-dinitro)-functionalized 1,2,3-triazole polyvalent metal salts remain poorly understood [10].

Herein, we report the rational design and synthesis of a novel tripotassium salt K_3_BNOT. Two gem-dinitro groups were introduced to enhance oxygen balance, and potassium ions were incorporated to construct a three-dimensional ionic coordination network for ultra-high crystal packing. A mild six-step synthetic route was developed with an overall yield of 19.1%, enabling gram-scale preparation. The crystal structure, intermolecular interactions, thermal stability, detonation performance, and structure-activity relationships were systematically investigated. This work expands the family of triazole-based energetic salts and provides new insights for the design of green lead-free energetic materials.

## 2. Results

### 2.1. Molecular Design and Optimization of the Synthetic Route

The molecular design in this study was based on two core principles. First, two gem-dinitro groups were introduced to maximize the oxygen balance and energy level of the 1,2,3-triazole parent scaffold [11,12]. Second, formation of the tripotassium salt was intended to construct an ionic three-dimensional coordination network, thereby enhancing crystal-packing density while also meeting the requirement for lead-free environmental compatibility. On this basis, a six-step synthetic route was developed (Scheme S1) using commercially available diaminomaleonitrile (DAMN) as the starting material. Initially, 4,5-dicyano-1,2,3-triazole (DCT) was prepared through a diazotization/cyclization reaction [13]. This intermediate was subsequently transformed into 4,5-bis(chloroximomethyl)-1,2,3-triazole (BCOT) through amidoxime addition followed by chlorination. Thereafter, gem-dinitro functionalization was accomplished using a dinitrogen pentoxide/chloroform nitration system, affording 4,5-bis(chloro(gem-dinitromethyl))-1,2,3-triazole (BNOT) [14,15]. Finally, the target compound 3K_3_BNOT·4H_2_O was obtained through two consecutive steps: dechlorination with potassium iodide and salt formation with potassium hydroxide.

The reaction conditions for the key steps were further optimized. In the nitration step, a mild dinitrogen pentoxide/chloroform system was employed, which avoided the strong corrosiveness and multiple side reactions commonly associated with traditional mixed-acid nitration systems and increased the reaction selectivity to 48%. In the salt-formation step, the crystallization rate was controlled using a methanol/water mixed-solvent system, enabling high-purity preparation of the target compound. The overall yield reached 19.1% based on DAMN, indicating the potential of this route for gram-scale synthesis. All intermediates were structurally characterized by IR, NMR, and elemental analysis, and the detailed data are provided in the Methods Section 4.

### 2.2. Regulatory Mechanism of Crystal Density by the Single-Crystal Structure and Coordination Network of 3K_3_BNOT·4H_2_O

High-quality single crystals of K_3_BNOT were obtained by slow evaporation at room temperature. Single-crystal X-ray diffraction analysis revealed that the hydrate 3K_3_BNOT·4H_2_O crystallizes in the triclinic crystal system with space group P-1 (Table 1). Partial positional disorder of the coordinated water molecules is observed in the asymmetric unit, which is a common phenomenon in hydrated energetic metal salts. The unit cell contains two independent asymmetric moieties with completely non-superimposable conformations, originating from the flexible coordination environment of K^+^ ions and the dense packing of anions and cations. The unit-cell parameters are a = 10.5811 (3) Å, b = 12.3754 (4) Å, c = 15.4412 (5) Å, α = 96.0000 (10)°, β = 97.2600 (10)°, and γ = 91.9050 (10)°, with a unit-cell volume of V = 1992.54 (11) Å^3^ and Z = 2 (corresponding to the two independent asymmetric moieties in one unit cell). The measured crystal density at 193 K reaches 2.077 g·cm^−3^, placing this compound among the highest-density triazole-based energetic potassium salts reported to date. The single-crystal data have been deposited in the Cambridge Crystallographic Data Centre (CCDC No. 1,491,887), and the complete crystallographic data are provided in Tables S1–S3 of the Supplementary Materials.

The comparison shows that the crystal density of 3K_3_BNOT·4H_2_O is more than 5% higher than that of the mainstream triazole-based energetic potassium salts reported to date, 15.0% higher than that of the classical high-energy explosive RDX, and 9.0% higher than that of HMX. These results clearly confirm the effectiveness of the molecular design strategy adopted in this study, in which regulation of a three-dimensional coordination network was used to achieve ultra-high crystal density.

**Figure 1:**
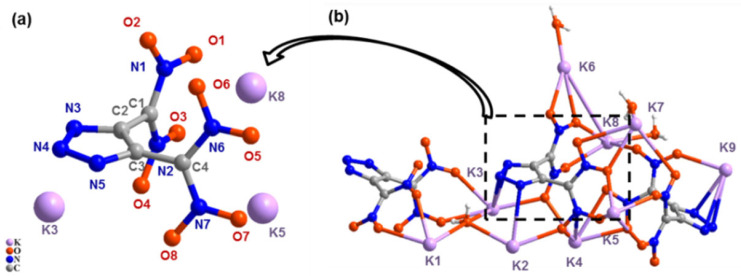
(**a**) Molecular structure of the compound; (**b**) the minimum asymmetric unit of the crystal. Arrows point to the enlarged framed structural regions.

The asymmetric unit of 3K_3_BNOT·4H_2_O consists of nine K^+^ cations, three 4,5-bis(gem-dinitromethyl)-1,2,3-triazolate anions, and four water molecules of crystallization (Figure 1). The N–N bond lengths in the triazole ring range from 1.340 to 1.343 Å, and the C–N bond length is 1.353 Å, indicating good aromaticity of the triazole ring.

As an alkali metal, K^+^ exhibits flexible coordination environments: K1, K4, and K7 are eight-coordinate, whereas K2, K3, K5, K6, K8, and K9 are nine-coordinate. The coordinating atoms originate from the O and N atoms of the anions and the O atoms of the crystal water. The K^+^ cations form coordination interactions with the O atoms of the gem-dinitro groups and the N atoms of the triazole ring (bond lengths: 2.613–3.466 Å). Through a μ_3_-bridging mode, these interactions generate one-dimensional K-O-K chains. Adjacent chains are linked by coordination interactions and crystal water, forming two-dimensional layers and further cross-linking into a three-dimensional ionic coordination lattice, which is a typical packing mode for alkali metal energetic salts.

**Figure 2:**
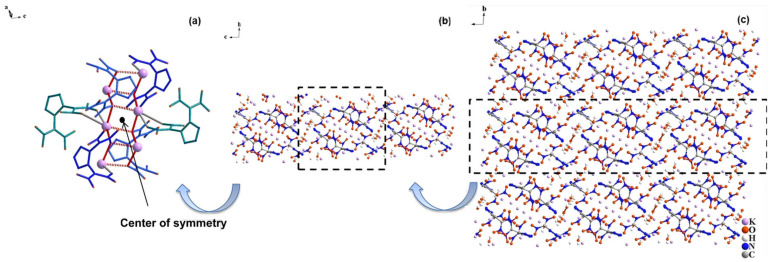
(**a**) Repeating Unit Structure; (**b**) 2D Structure of Each Layer; (**c**) Crystal Packing Diagram. Arrows point to the enlarged framed structural regions.

Structure–activity relationship analysis indicates that this three-dimensional ionic coordination network is the key factor responsible for the ultra-high crystal density (Figure 2). On the one hand, the strong K-O/K-N coordination bonds significantly shorten the distances between anions and cations, reduce the void volume within the unit cell, and improve crystal-packing efficiency [16,17]. On the other hand, the gem-dinitro anions act as multidentate ligands and enable dense packing within the unit cell through multidirectional coordination, thereby avoiding the loose packing commonly observed in traditional energetic materials dominated by weak intermolecular interactions [18,19]. These results demonstrate that the precise construction of polyvalent metal coordination networks can effectively enhance the crystal density of energetic materials and provides a feasible strategy for overcoming the bottleneck of simultaneously achieving high density and high energy [16,20].

### 2.3. Analysis of Intermolecular Interactions and Electronic Structure

**Figure 3:**
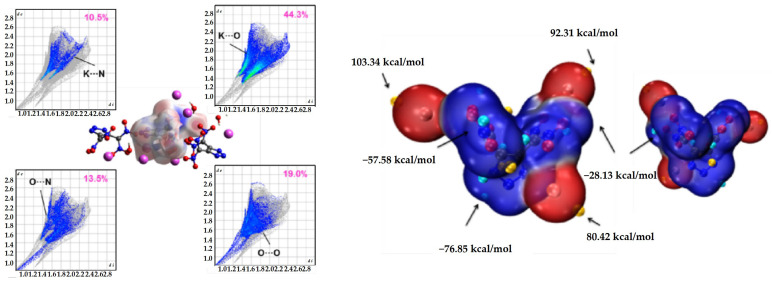
TD Fingerprint Plots and Hirshfeld Surface Maps of 3K_3_BNOT·4H_2_O.

To gain deeper insight into the influence of intermolecular interactions on the macroscopic properties of the material, Hirshfeld surface analysis and two-dimensional fingerprint plot analysis of the anion in 3K_3_BNOT·4H_2_O were performed using CrystalExplorer 17.5 [21,22] (Figure 3). The results show that K···O interactions account for 44.3% of the total, whereas K···N interactions contribute 10.5%. Together, these two types of interactions represent 54.8% of the total and therefore dominate the intermolecular interaction pattern of the system. In comparison, O···O interactions account for 19.0%, O···N interactions for 13.5%, and all other interactions make only minor contributions.

Mechanistic analysis indicates that the red regions on the Hirshfeld surface are mainly concentrated at the contact sites between K+ and the O and N atoms, confirming that strong ion–dipole interactions are the dominant intermolecular force in the system [23]. By contrast, hydrogen-bonding interactions contribute only a very small proportion, which is fully consistent with the structural characteristics of the three-dimensional coordination network. This interaction mode, dominated by strong coordination interactions, not only improves crystal-packing density but also stabilizes the framework structure, thereby providing a structural basis for the thermal stability of the material [24,25].

### 2.4. Thermal Decomposition Behavior and Kinetic Mechanism

The thermal decomposition behavior of 3K_3_BNOT·4H_2_O was investigated by differential scanning calorimetry (DSC) over the temperature range of 50–500 °C at a heating rate of 10 °C·min^−1^. The results show that no obvious melting endotherm is observed before thermal decomposition, indicating that 3K_3_BNOT·4H_2_O undergoes direct solid-state decomposition. The initial decomposition temperature, peak decomposition temperature, and final decomposition temperature are 171.2 °C, 183.8 °C, and 196.4 °C, respectively. In addition, the decomposition peak is sharp and narrow, suggesting a rapid decomposition rate and concentrated energy release (Table 2). This behavior is consistent with the thermal characteristics of primary explosives.

**Figure 4:**
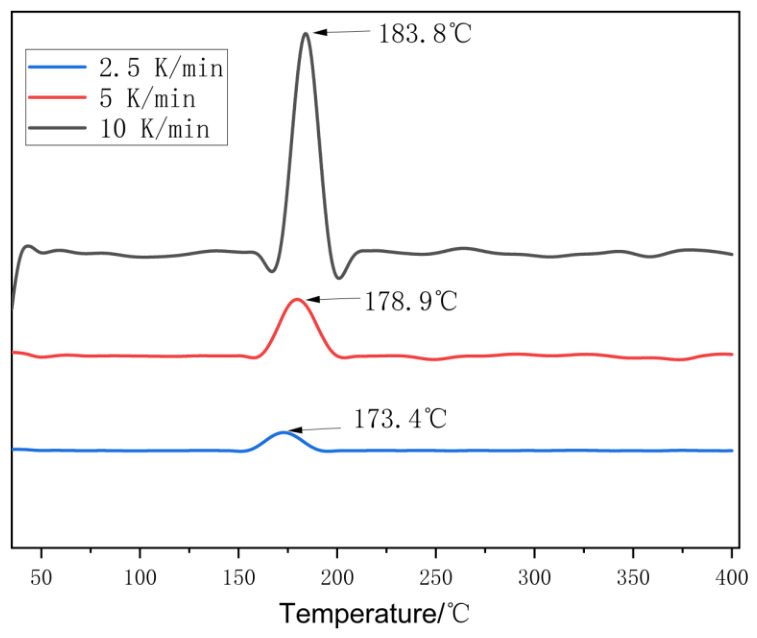
DSC of Compound 3K_3_BNOT·4H_2_O.

The key application requirement for primary explosives is not exceptionally high thermal stability, but the ability to ensure thermal safety during storage at ambient temperature while allowing rapid and concentrated energy release after ignition. For the mainstream lead-free primary explosives currently used in industrial practice, the suitable range of peak decomposition temperature is 160–250 °C (Figure 4). The 3K_3_BNOT·4H_2_O prepared in this study exhibits a peak decomposition temperature of 183.8 °C, which falls well within this practical application window. This temperature is sufficiently high to avoid inadequate storage stability caused by premature decomposition, yet not so high as to induce problems such as insufficient initiation sensitivity or ignition difficulty. Therefore, 3K_3_BNOT·4H_2_O is well matched to the application requirements of lead-free primary explosives and does not exhibit the practical limitation of “insufficient thermal stability.

To further clarify its thermal decomposition mechanism, non-isothermal DSC measurements were carried out at heating rates of 2.5, 5, and 10 °C·min^−1^. The results show that the decomposition peak temperature gradually increases from 173.4 °C to 183.8 °C with increasing heating rate, which is consistent with the typical non-isothermal decomposition behavior of energetic materials. The thermal decomposition data were subsequently analyzed kinetically using the Kissinger method, the Ozawa method, and the model-free Friedman method, and the resulting kinetic parameters are summarized in Table 3.

The difference in activation energy calculated by the Kissinger method and the Ozawa method is 25.6 kJ·mol^−1^, corresponding to a relative deviation of 11.6%. This value falls within the reasonable error range for kinetic analysis of the thermal decomposition of energetic materials, thereby confirming the reliability of the fitted results. Both methods give relatively high activation energies, indicating that 3K_3_BNOT·4H_2_O possesses a high energy barrier for thermal decomposition and therefore good thermal stability.

**Figure 5:**
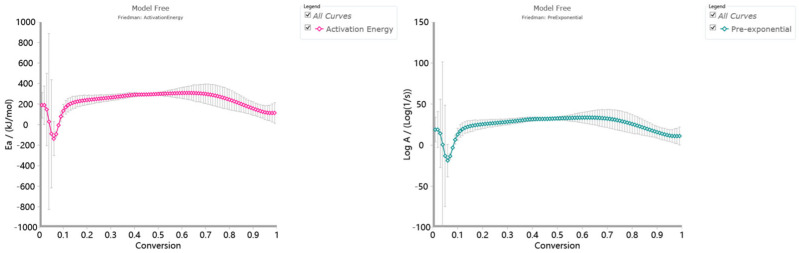
(**a**) Plot of Activation Energy versus Conversion; (**b**) Plot of Pre-exponential Factor versus Conversion.

The variation in activation energy and pre-exponential factor over the conversion range α = 0.01–0.99 was further analyzed using the Friedman method in Figure 5. The results show that, within the main decomposition interval of α = 0.1–0.9, the activation energy remains at a relatively high level of 180–220 kJ·mol^−1^. The pre-exponential factor lgA increases to a maximum value of 23.535 at α = 0.1–0.15 and then gradually decreases with increasing conversion. This trend is consistent with the change in reaction rate during thermal decomposition. In the initial stage, the rapid increase in the pre-exponential factor corresponds to the rapid onset of decomposition and accelerated energy release. During the main decomposition stage, the combination of high activation energy and a high pre-exponential factor ensures stable and concentrated energy release, which is consistent with the energy-release requirements of primary explosives.

Mechanistic analysis indicates that the relatively high thermal decomposition energy barrier of 3K_3_BNOT·4H_2_O originates from the stabilizing effect of its three-dimensional ionic coordination network on the molecular framework. The strong K-O/K-N coordination bonds restrict the thermal motion of the energetic groups and increase the energy barrier of the decomposition process, whereas the rapid decomposition characteristics of the gem-dinitro groups ensure concentrated energy release. These results clarify the regulatory mechanism by which the coordination network governs the thermal decomposition behavior of the material and provide a new perspective for optimizing the thermal stability of energetic materials.

### 2.5. Detonation Performance and Structure–Activity Relationship Analysis

Using the measured crystal density of 2.077 g·cm^−3^ at 25 °C, the heat of formation of 3K_3_BNOT·4H_2_O was calculated to be −135.3 kJ·mol^−1^ by the Born–Haber cycle. On the basis of the measured density and the calculated heat of formation, the detonation parameters of 3K_3_BNOT·4H_2_O were calculated with the EXPLO5 program and compared with those of classical energetic materials. The results are summarized in Table 4 [25,26].

The calculations show that 3K_3_BNOT·4H_2_O has a detonation velocity of 8836 m·s^−1^ and a detonation pressure of 28.6 GPa [27]. Its detonation velocity is markedly higher than that of the classical high-energy explosive RDX and approaches that of HMX. Notably, the crystal density of 3K_3_BNOT·4H_2_O is 15.0% higher than that of RDX, which is a key factor contributing to its excellent detonation performance (see Supplementary Materials).

The high density of 3K_3_BNOT·4H_2_O originates from the high mass fraction of K^+^ cations and the dense ionic lattice packing. Its detonation velocity is higher than that of RDX, showing good energy potential.

## 3. Discussion

3K_3_BNOT·4H_2_O is a completely lead-free energetic salt hydrate, with no heavy-metal elements in its structure or preparation process. Its detonation products are dominated by environmentally benign gases (N_2_, CO_2_), eliminating the environmental and health hazards of traditional lead-based explosives.

The peak decomposition temperature of 183.8 °C is within the suitable range for energetic materials, balancing storage stability and energy release. The three-dimensional ionic coordination lattice contributes to dense packing and thermal stability, which is a common stabilization mechanism for alkali metal energetic salts.

The synthetic route uses commercially available raw materials with mild conditions, enabling gram-scale preparation, which supports further research on its application potential as a green energetic material. This compound has structural potential as a high-energy primary explosive, and its initiating performance needs further experimental verification.

## 4. Materials and Methods

### 4.1. Reagents and Instruments

Diaminomaleonitrile (96%), sodium nitrite (99%), hydroxylamine hydrochloride, potassium iodide, and potassium hydroxide were purchased from Shanghai Aladdin Biochemical Technology Co., Ltd. (Shanghai, China). All solvents were of analytical grade and used without further purification.

Instruments: AV 500 NMR spectrometer (Bruker, Fällanden, Switzerland), NEXUS 870 FT-IR spectrometer (Nicolet, Madison, WI, USA), Vario-EL-3 elemental analyzer (Elementar, Frankfurt, Germany), DSC 204 differential scanning calorimeter (NETZSCH, Selb, Germany), D8 Venture single-crystal X-ray diffractometer (Bruker, Karlsruhe, Germany), BAM fall-hammer apparatus, BAM friction apparatus (Bundesanstalt für Materialforschung und -prüfung, Berlin, Germany).

### 4.2. General Synthetic Procedures

The detailed synthetic procedures for intermediates DCT, BCOT, BNOT, K_2_BNOT, and the final product 3K_3_BNOT·4H_2_O are provided in the Supplementary Materials. Anhydrous 3K_3_BNOT·4H_2_O was obtained by vacuum drying the hydrate product at 120 °C under 2 mbar for 24 h.

### 4.3. Characterization Methods

IR spectroscopy: KBr pellets, 4000–400 cm^−1^.

NMR spectroscopy: ^13^C NMR at 125 MHz in DMSO-d_6_.

Elemental analysis: Determined for C, H, N using a Vario-EL-3 elemental analyzer.

SC-XRD: Data collected at 193 K using CuKα radiation, solved by direct methods and refined by full-matrix least-squares on F^2^ using SHELXL-2018.

DSC: 50–500 °C, heating rates of 2.5, 5, and 10 °C·min^−1^, N_2_ atmosphere (50 mL·min^−1^).

### 4.4. Synthesis of Derivatives

#### 4.4.1. Synthesis of 4,5-Dicyano-1,2,3-triazole (DCT)

A solution of diaminomaleonitrile (DAMN, 10.81 g, 100.0 mmol) in deionized water (250 mL) was prepared, and 100 mL of 1 M hydrochloric acid was added slowly while maintaining the temperature below 5 °C using an ice bath. After the addition was complete, the reaction mixture was cooled to below 0 °C using an ice–salt bath, and sodium nitrite (6.89 g, 100.0 mmol) was added slowly in portions over 30 min. The reaction mixture was then stirred at 0 °C for 1 h, removed from the ice–salt bath, and allowed to warm gradually to room temperature, where it was stirred for an additional 4 h.

After completion of the reaction, the black insoluble solid was removed by filtration. The filtrate was extracted five times with diethyl ether (total volume: 600 mL). The combined organic phases were washed with deionized water to neutrality, dried over anhydrous magnesium sulfate, filtered, and concentrated under reduced pressure to induce crystallization. The resulting yellow crystals were collected by filtration and dried under vacuum to afford DCT (10.60 g, 89% yield).

Characterization data for DCT:IR (KBr, cm^−1^): 3261 (N–H), 2263 (C≡N), 1383, 1130, 792 (triazole ring). ^1^H NMR (DMSO-d_6_, 500 MHz): δ = 7.63 (s, 1H) ppm. ^13^C NMR (DMSO-d_6_, 125 MHz): δ = 124.4, 110.8 ppm. Elemental analysis calcd for C_4_HN_4_ (%): C 41.40, H 0.87, N 57.73; found: C 41.37, H 0.90, N 57.70.

#### 4.4.2. Synthesis of 4,5-Bis(chloroximomethyl)-1,2,3-triazole (BCOT)

4,5-Dicyano-1,2,3-triazole (DCT, 9.52 g, 80.0 mmol) was added to deionized water (100 mL). Hydroxylamine hydrochloride (13.90 g, 200.0 mmol) was then added at room temperature, followed by the slow portionwise addition of sodium carbonate (10.60 g, 100.0 mmol), during which substantial gas evolution was observed. After the pH was adjusted to 8–9, the reaction mixture was heated to 60 °C and stirred for 2 h.

The reaction was then terminated, and after cooling to room temperature, the resulting white solid was collected by filtration, washed with deionized water (50 mL), and dried under vacuum. This white solid was then added, under a low-temperature bath at −15 °C, to a mixed solution of deionized water (100 mL) and concentrated hydrochloric acid (100 mL). With vigorous stirring, 35 mL of an aqueous sodium nitrite solution containing NaNO_2_ (13.80 g, 200 mmol) was added dropwise while maintaining the reaction temperature below −10 °C.

After the addition was complete, the reaction mixture was left to stand at room temperature overnight until no further brown fumes were evolved. The mixture was then extracted five times with anhydrous diethyl ether (total volume: 600 mL). The combined organic phases were washed with deionized water to neutrality, dried over anhydrous magnesium sulfate, filtered, and concentrated under reduced pressure to remove the solvent, affording BCOT as a yellow solid (10.3 g, 57% yield).

Characterization data for BCOT:IR (KBr, cm^−1^): 3438 (O–H), 3333 (–NH_2_), 3102 (N–H), 1684 (C=N), 1125, 996, 824 (triazole ring). ^1^H NMR (DMSO-d_6_, 500 MHz): δ = 9.571 (s, 2H, –OH), 6.732 (s, 4H, –NH_2_) ppm. ^13^C NMR (DMSO-d_6_, 125 MHz): δ = 146.79 [–C(NOH)NH_2_], 133.28 (triazole ring C) ppm. Elemental analysis calcd for C_4_H_3_Cl_2_N_5_O_2_ (%): C 18.08, H 1.14, N 33.46; found: C 18.05, H 1.17, N 33.49.

#### 4.4.3. Synthesis of 4,5-Bis(chloro(gem-dinitromethyl))-1,2,3-triazole (BNOT)

Dinitrogen pentoxide (2.20 g, 20 mmol) was added to chloroform (50 mL) at 0 °C, and a chloroform suspension of BCOT (1.34 g, 6 mmol) was then added dropwise over 15 min. After the addition was complete, the reaction mixture was heated to 45 °C and stirred for 1 h.

The white insoluble solid was removed by filtration, and the filtrate was concentrated under reduced pressure to give a yellow oily material. The crude product was purified by column chromatography on silica gel using ethyl acetate/hexane (1:3, *v*/*v*) as the eluent, affording BNOT as a yellow oil (1.0 g, 48% yield).

Characterization data for BNOT:IR (KBr, cm^−1^): 3261 (O–H), 3035 (N–H), 1617 (C=N), 1570, 1302 (–NO_2_), 1125, 996, 824 (triazole ring), 895 (C–Cl). ^1^H NMR (DMSO-d_6_, 500 MHz): δ = 12.815 (s, 1H, N–H) ppm. ^13^C NMR (DMSO-d_6_, 125 MHz): δ = 138.52 [–C(NO_2_)_2_Cl], 126.52 (triazole ring C) ppm. Elemental analysis calcd for C_4_HCl_2_N_7_O_8_ (%): C 11.84, H 0.25, N 29.93; found: C 11.81, H 0.28, N 29.96.

#### 4.4.4. Synthesis of Tripotassium 4,5-Bis(gem-dinitromethyl)-2H-1,2,3-triazolate (K_3_BNOT)

BNOT (1.00 g, 2.8 mmol) was dissolved in anhydrous methanol (20 mL), and a solution of potassium iodide (3.00 g, 18.1 mmol) in methanol (30 mL) was added dropwise at room temperature. The reaction mixture rapidly turned dark purple-black. It was then allowed to react overnight at room temperature, during which a yellow solid precipitated.

The solid was collected by filtration and washed successively with methanol (5 mL) and diethyl ether (5 mL), affording K_2_BNOT as a yellow solid. Then it was suspended in methanol (20 mL). After the addition of 2 mL of deionized water, the mixture was heated to 50 °C until complete dissolution was achieved. Solid potassium hydroxide (0.28 g, 5 mmol) was then added slowly in portions over 10 min. After complete dissolution, the reaction mixture was stirred at room temperature for 4 h.

The reaction solution was concentrated under reduced pressure to approximately 5 mL, and the resulting yellow crystals were collected by filtration, washed with cold methanol (2 mL), and dried under vacuum to afford the hydrate form of K_3_BNOT (0.44 g, 80.3% yield).

#### 4.4.5. Preparation of 3K_3_BNOT·4H_2_O

The hydrate product was placed in a vacuum oven and dried at 120 °C under 1×10^−3^ mmHg for 24 h to obtain 3K_3_BNOT·4H_2_O. All performance tests (DSC, sensitivity, detonation calculations) were performed using this sample.

Characterization data for K_3_BNOT:IR (KBr, cm^−1^): 3608, 3499, 2346; 1626, 1478 (C=N); 1571, 1358 (–NO_2_); 1304, 1266 (C–N); 1162 (N–O); 815 (triazole ring); 782; 610, 988.^13^C NMR (DMSO-d_6_, 125 MHz): δ = 161.13 [–C(NO_2_)_2_^−^], 110.00 (triazole ring C) ppm. Elemental analysis calcd for K_3_C_4_N_7_O_8_ (%): C 9.96, H 0, N 20.43; found: C 9.93, H 0.03, N 20.46.

## 5. Conclusions

A novel bis(gem-dinitro)-substituted 1,2,3-triazole tripotassium salt 3K_3_BNOT·4H_2_O was rationally designed and synthesized via a mild six-step route. Through the construction of a three-dimensional K-O/K-N coordination network, K_3_BNOT achieves an ultra-high crystal density of 2.077 g·cm^−3^ at 193 K, which is 15.0% higher than that of RDX. It exhibits suitable thermal stability (183.8 °C), moderate sensitivity (IS = 5 J, FS = 60 N), and detonation performance superior to RDX. As a completely lead-free system, K_3_BNOT eliminates the environmental and health hazards of traditional lead-based primary explosives and shows great potential for industrial applications. Future work will focus on optimizing the thermal stability of 3K_3_BNOT·4H_2_O through metal ion regulation and systematically evaluating its practical initiation performance via professional testing platforms.

## Figures and Tables

**Table 1:** Crystallographic Data of 3K_3_BNOT·4H_2_O.

Empirical Formula	C_12_H_8_K_9_N_21_O_28_
Formula weight	1246.29
Temperature/K	193.00
Crystal system	triclinic
Space group	P-1
a/Å	10.5811 (3)
b/Å	12.3754 (4)
c/Å	15.4412 (5)
α/°	96.0000 (10)
β/°	97.2600 (10)
γ/°	91.9050 (10)
Volume/Å3	1992.54 (11)
Z	2
ρ g/cm^3^	2.077
μ/mm^−1^	9.820
F(000)	1244.0
Crystal size/mm^3^	0.13 × 0.11 × 0.09
Radiation	CuKα (λ = 1.54178)
2Θ range for data collection/°	5.804 to 136.8
Index ranges	−12 ≤ h ≤ 12, −14 ≤ k ≤ 14, −18 ≤ l ≤ 18
Reflections collected	28,229
Independent reflections	7238 [Rint = 0.0418, Rsigma = 0.0349]
Data/restraints/parameters	7238/51/695
Goodness-of-fit on F2	1.275
Final R indexes [I ≥ 2σ (I)]	R1 = 0.0446, wR2 = 0.0965
Final R indexes [all data]	R1 = 0.0497, wR2 = 0.0974
Largest diff. peak/hole/e Å^−3^	1.07/−0.42

CCDC deposition number: 1491887.

**Table 2:** Comparison of Peak Thermal Decomposition Temperatures between K_3_BNOT and Typical Energetic Primary Materials.

Compound Name	Peak Thermal Decomposition Temperature/°C	Compound Type
3K_3_BNOT·4H_2_O (this work)	183	1,2,3-Triazole-Based Energetic Potassium Salt
Potassium 4,5-dinitro-1,2,3-triazolate	176	1,2,3-Triazole-Based Energetic Potassium Salt
Potassium 3-(gem-dinitromethyl)-1,2,4-triazolate	192	1,2,4-Triazole-Based Energetic Potassium Salt
Lead styphnate (LTNR)	282	Lead-based primary explosive
Lead azide (LA)	350	Lead-based primary explosive

**Table 3:** Thermal Decomposition Kinetic Parameters of 3K_3_BNOT·4H_2_O.

Kinetic Method	Correlation Coefficient (R2)	Activation Energy, Ea/(kJ·mol^−1^)	Pre-Exponential Factor, lgA/(s^−1^)
Kissinger Method	0.963	220.3	16.92
Ozawa Method	0.958	194.7	15.87
Friedman Method	0.995	194.8	21.51

**Table 4:** Performance Comparison between 3K_3_BNOT·4H_2_O and Classical Energetic Materials.

Compound	Crystal Density/ (g·cm^−3^)	Detonation Velocity/ (m·s^−1^)	Detonation Pressure/ GPa	Impact Sensitivity/J	Friction Sensitivity/N
3K_3_BNOT·4H_2_O	2.077	8836	28.6	5.0	60
RDX	1.806	8795	34.9	7.5	120
HMX	1.905	9100	39.3	7.4	120

Impact and friction sensitivities were measured by standard BAM methods: impact sensitivity using a 2 kg drop weight (20 mg sample, 10 parallel tests); friction sensitivity using a porcelain rod (10 mg sample, 10 parallel tests), both at 50% ignition probability.

## Data Availability

The original contributions presented in this study are included in the article and its Supplementary Materials. The crystallographic data for the compound 3K_3_BNOT·4H_2_O have been deposited in the Cambridge Crystallographic Data Centre (CCDC) under deposition number 1,491,887. Further inquiries can be directed to the corresponding author.

## Supplementary Materials

The following supporting information can be downloaded at: https://www.mdpi.com/article/10.3390/molecules31121992/s1; Scheme S1: Six-step synthetic route of tripotassium 4,5-bis(gem-dinitromethyl)-2H-1,2,3-triazolate (3K_3_BNOT·4H_2_O); Figure S1: FT-IR spectrum of 4,5-dicyano-1,2,3-triazole (DCT); Figure S2: ^1^H NMR spectrum of DCT (500 MHz, DMSO-d_6_); Figure S3: ^13^C NMR spectrum of DCT (125 MHz, DMSO-d_6_); Figure S4: FT-IR spectrum of 4,5-bis(chloroximomethyl)-1,2,3-triazole (BCOT); Figure S5: ^1^H NMR spectrum of BCOT (500 MHz, DMSO-d_6_); Figure S6: ^13^C NMR spectrum of BCOT (125 MHz, DMSO-d_6_); Figure S7: FT-IR spectrum of 4,5-bis(chloro(gem-dinitromethyl))-1,2,3-triazole (BNOT); Figure S8: ^1^H NMR spectrum of BNOT (500 MHz, DMSO-d_6_); Figure S9: ^13^C NMR spectrum of BNOT (125 MHz, DMSO-d_6_); Figure S10: Full FT-IR spectrum of K_3_BNOT (4000–400 cm^−1^); Figure S11: Full ^13^C NMR spectrum of K_3_BNOT (125 MHz, DMSO-d_6_); Table S1: Atomic coordinates and equivalent isotropic displacement parameters of 3K_3_BNOT·4H_2_O; Table S2: Detailed parameters of detonation performance calculations (EXPLO5 v6.05); File S1: Crystallographic information file (CIF) for 3K_3_BNOT·4H_2_O (CCDC No. 1,491,887).

## Abbreviations

The following abbreviations are used in this manuscript:

BAM	Bundesanstalt für Materialforschung und -prüfung
BCOT	4,5-Bis(chloroximomethyl)-1,2,3-triazole
BNOT	4,5-Bis(chloro(gem-dinitromethyl))-1,2,3-triazole
CCDC	Cambridge Crystallographic Data Centre
DAMN	Diaminomaleonitrile
DFT	Density Functional Theory
DSC	Differential scanning calorimetry
ESP	Electrostatic potential
FT-IR	Fourier-transform infrared spectroscopy
FS	Friction sensitivity
HMX	Octahydro-1,3,5,7-tetranitro-1,3,5,7-tetrazocine
IR	Infrared spectroscopy
IS	Impact sensitivity
K2BNOT	Dipotassium 4,5-bis(chloro(gem-dinitromethyl))-1,2,3-triazolate
K3BNOT	Tripotassium 4,5-bis(gem-dinitromethyl)-2H-1,2,3-triazolate
LA	Lead azide
LTNR	Lead styphnate
NMR	Nuclear magnetic resonance
RDX	Hexahydro-1,3,5-trinitro-1,3,5-triazine
SC-XRD	Single-crystal X-ray diffraction
