# Supplementary material for: Ultra-High-Density Tripotassium 4,5-Bis(gem-dinitromethyl)-1,2,3-triazolate Hydrate (3K3BNOT·4H2O): A Lead-Free Triazole-Based Energetic Salt
Source: Molecules. 2026 Jun 7;31(12):1992. doi: 10.3390/molecules31121992 (PMC13304913; doi:10.3390/molecules31121992)
Supplement: Supplementary file 1 [file molecules-31-01992-s001.zip › molecules-4289228-supplementary.pdf]

Supporting Information

Ultra-High-Density Tripotassium 4,5-Bis(gem-dinitromethyl)-1,2,3-triazolate Hydrate ($3K_3BNOT{\cdot}4H_2O$): A Lead-Free Triazole-Based Energetic Salt

Ruokai Pei[1], Yang Wu[1] and Yinglei Wang[1, 2] *

[1] Institute of Energetic Materials, Xi'an Modern Chemistry Research Institute, Xi'an 710065, China; p18955883901@163.com (R.P.); wuy_204@163.com (Y.W.)
[2] State Key Laboratory of Fluorine & Nitrogen Chemicals, Xi'an 710065, China
* Correspondence: wangyl204@163.com

S1. Synthetic Route

Scheme S1. Six-step synthetic route of tripotassium 4,5-bis(gem-dinitromethyl)-2H-1,2,3-triazolate ($3K_3BNOT{\cdot}4H_2O$)

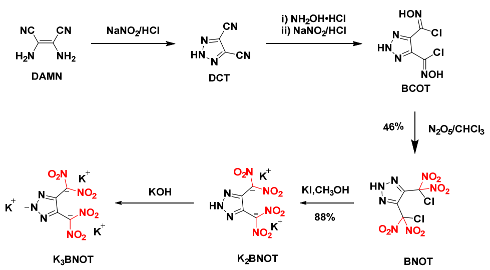

S2. Full Spectral Data of All Intermediates and Target Compound

Figure S1. FT-IR spectrum of 4,5-dicyano-1,2,3-triazole (DCT)

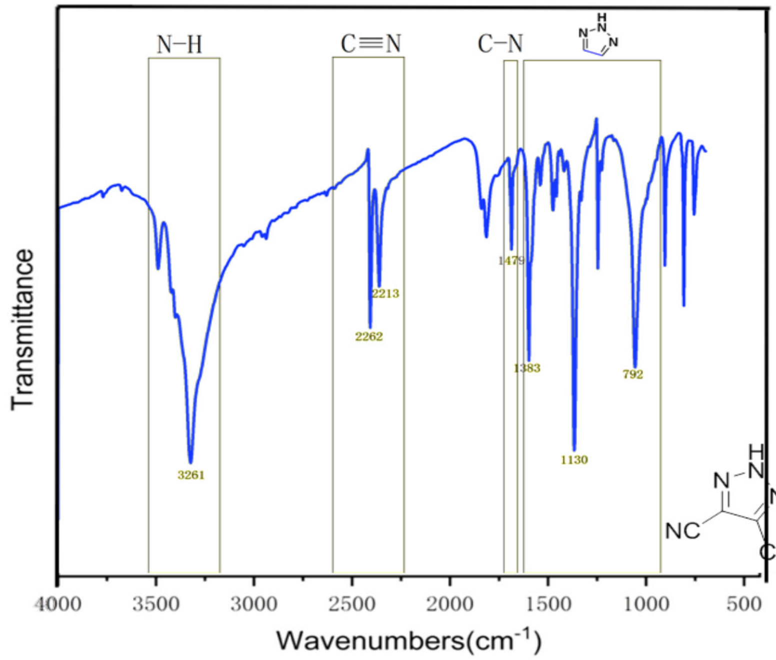

Figure S2. ^{1}H NMR spectrum of DCT (500 MHz, DMSO-d$_6$)

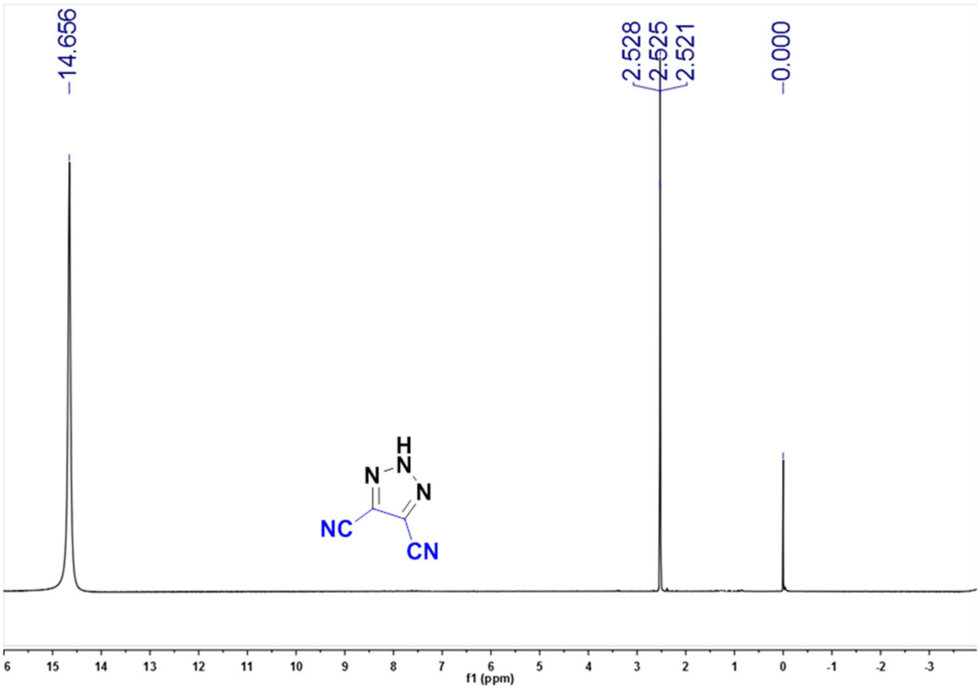

Figure S3. ^{13}C NMR spectrum of DCT (125 MHz, DMSO-d$_6$)

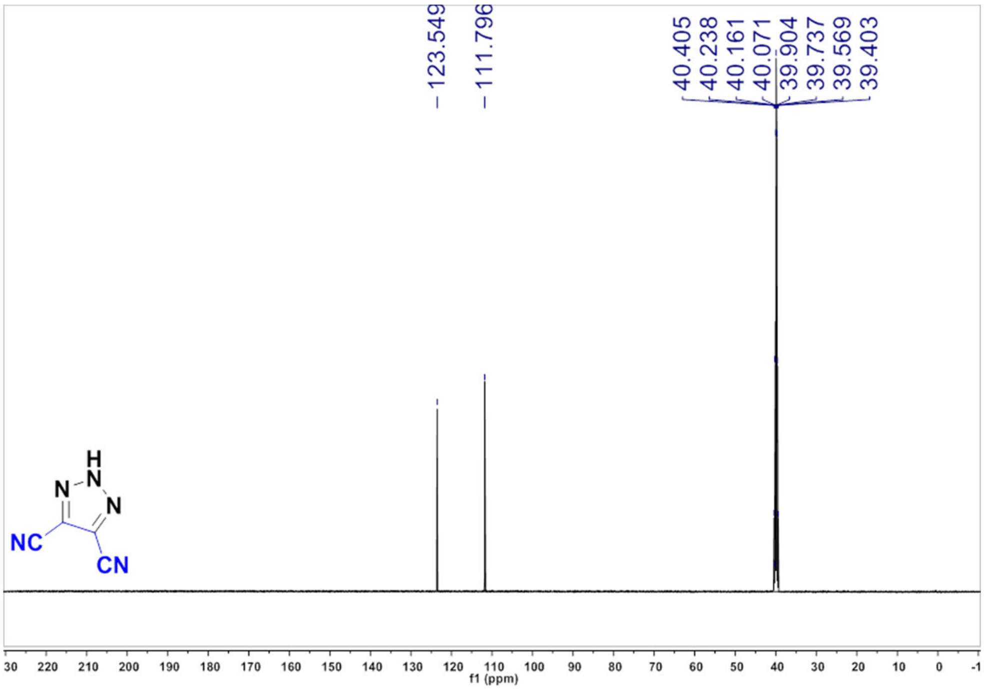

Figure S4. FT-IR spectrum of 4,5-bis(chloroximomethyl)-1,2,3-triazole (BCOT)

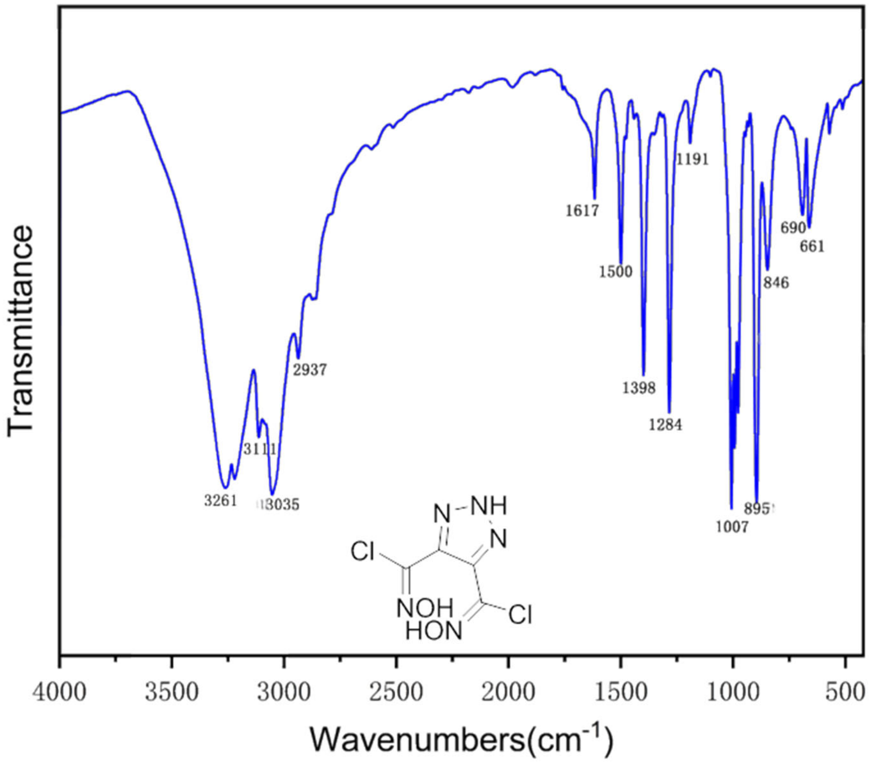

Figure S5. ^{1}H NMR spectrum of BCOT (500 MHz, DMSO-d_6)

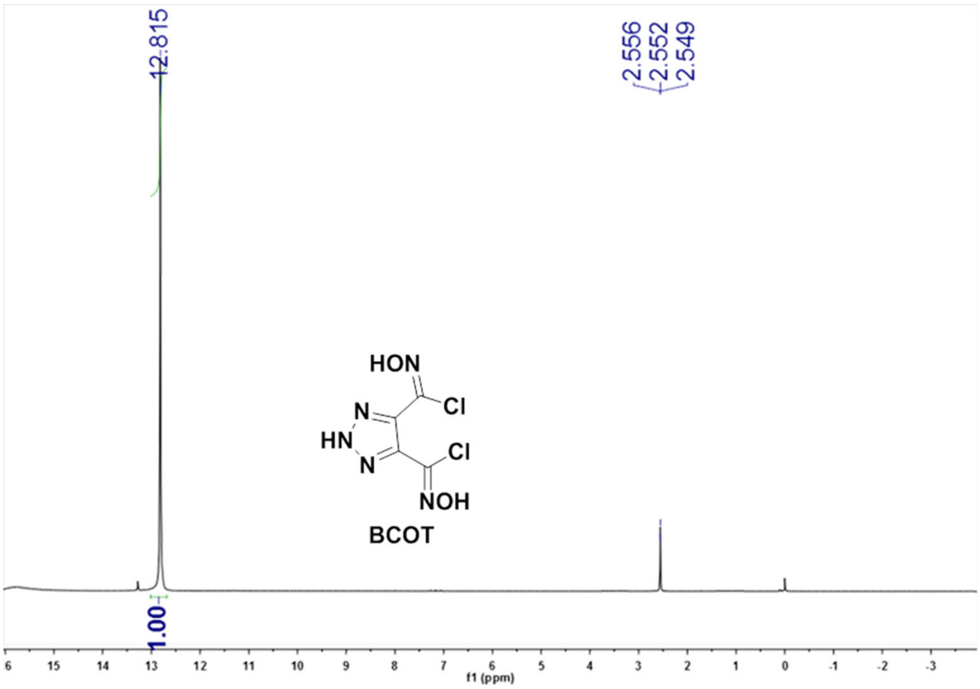

Figure S6. ^{13}C NMR spectrum of BCOT (125 MHz, DMSO-d_6)

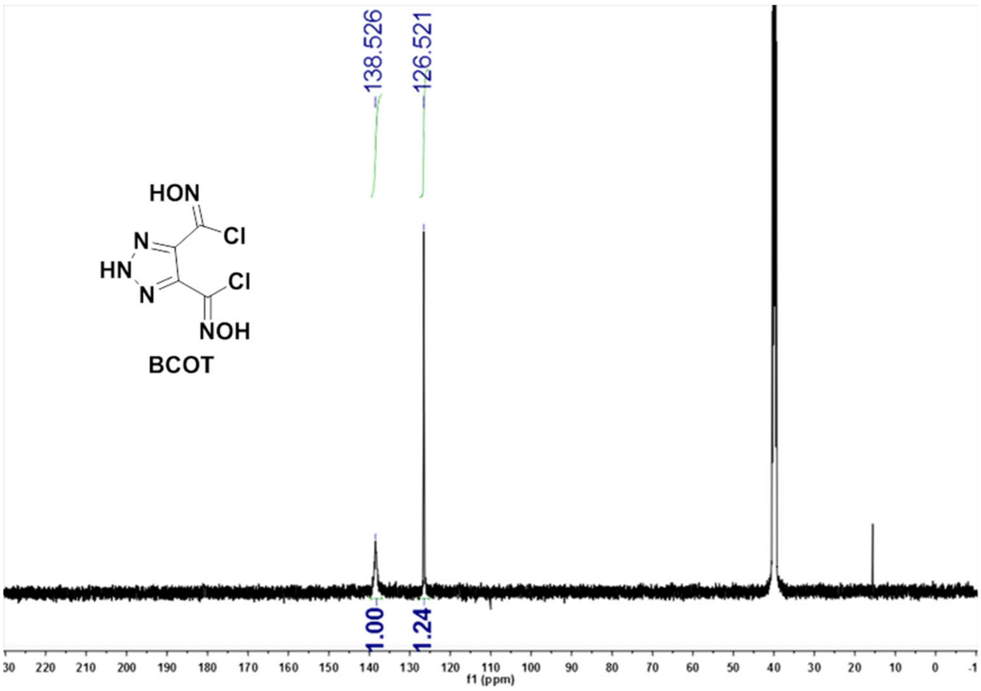

Figure S7. FT-IR spectrum of 4,5-bis(chloro(gem-dinitromethyl))-1,2,3-triazole (BNOT)

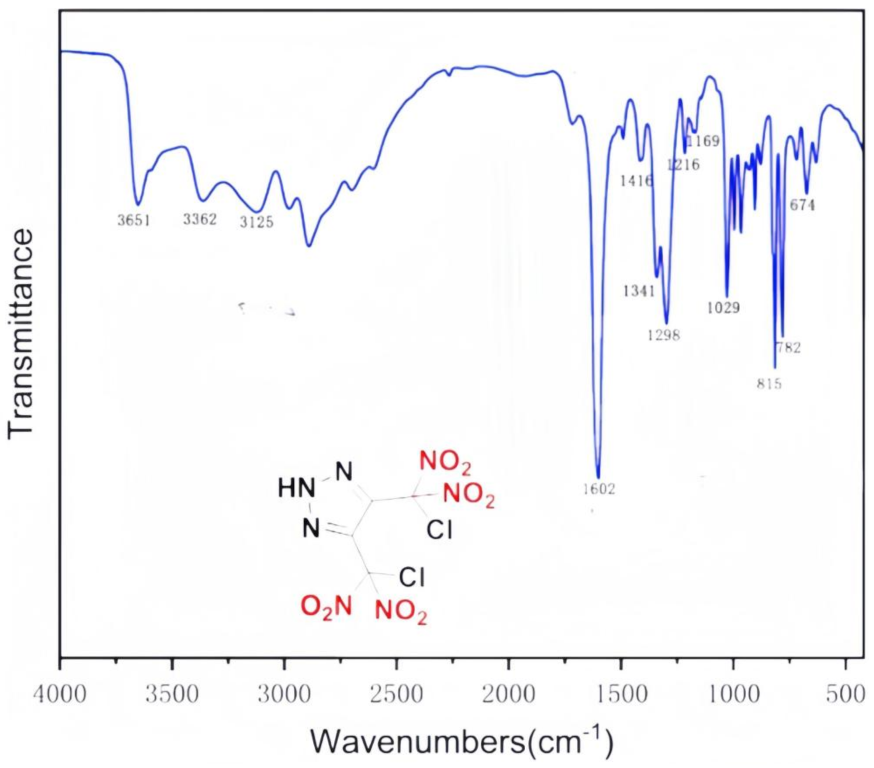

Figure S8. ^{1}H NMR spectrum of BNOT (500 MHz, DMSO-d_6)

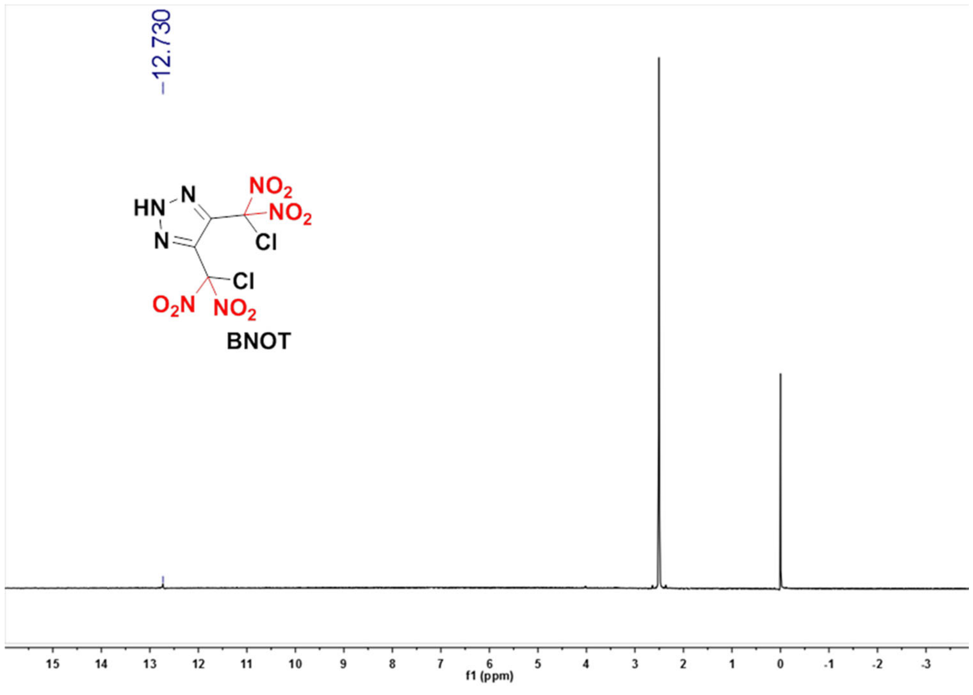

Figure S9. ^{13}C NMR spectrum of BNOT (125 MHz, DMSO-d_6)

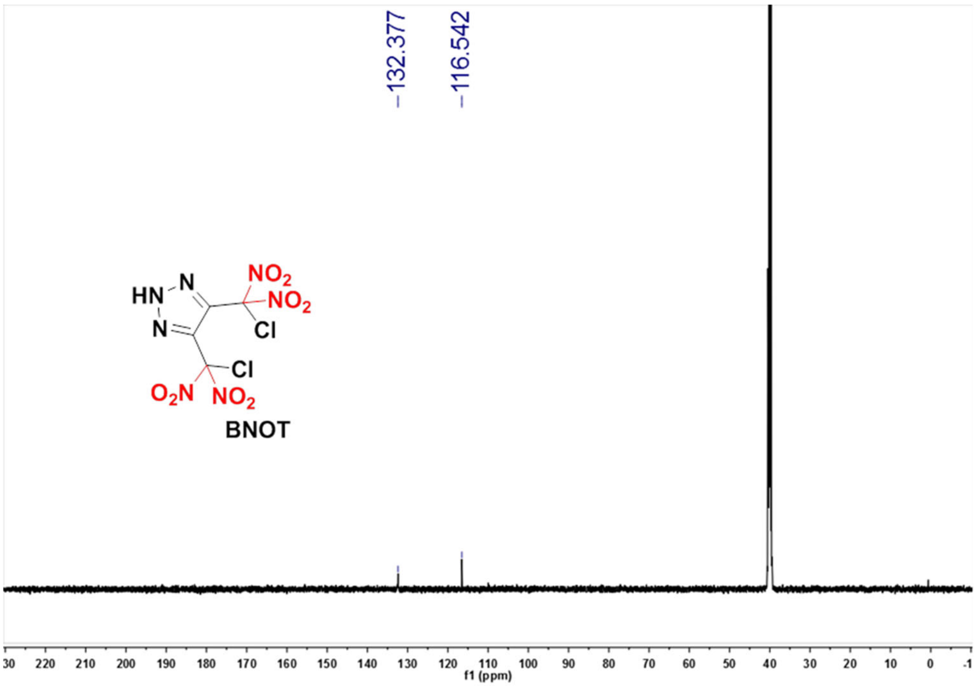

Figure S10. Full FT-IR spectrum of anhydrous $3K_3BNOT{\cdot}4H_2O$ (4000–400 cm^{-1})

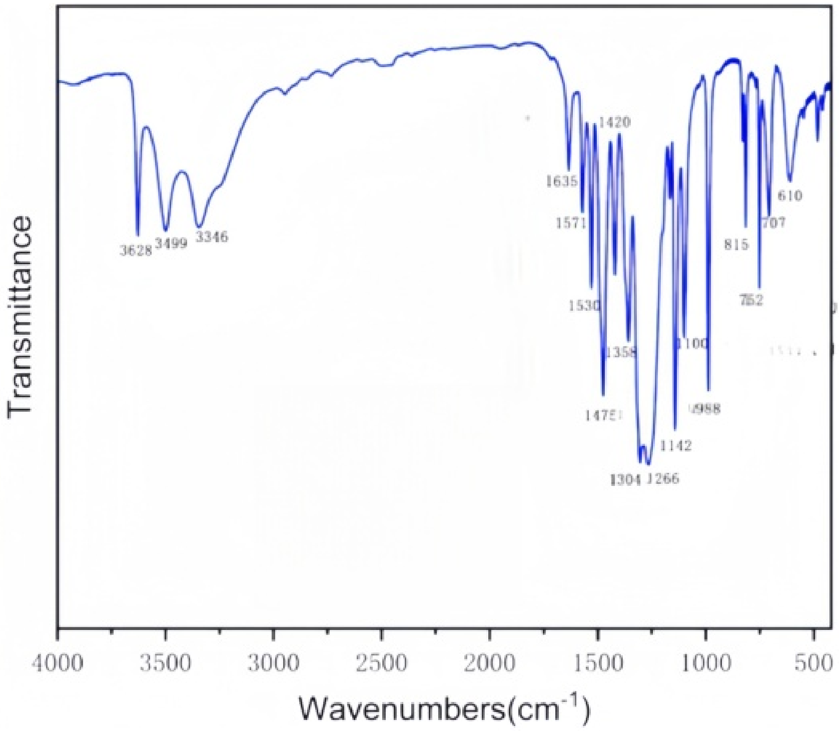

Figure S11. Full ^{13}C NMR spectrum of anhydrous $3K_3BNOT{\cdot}4H_2O$ (125 MHz, DMSO-d_6)

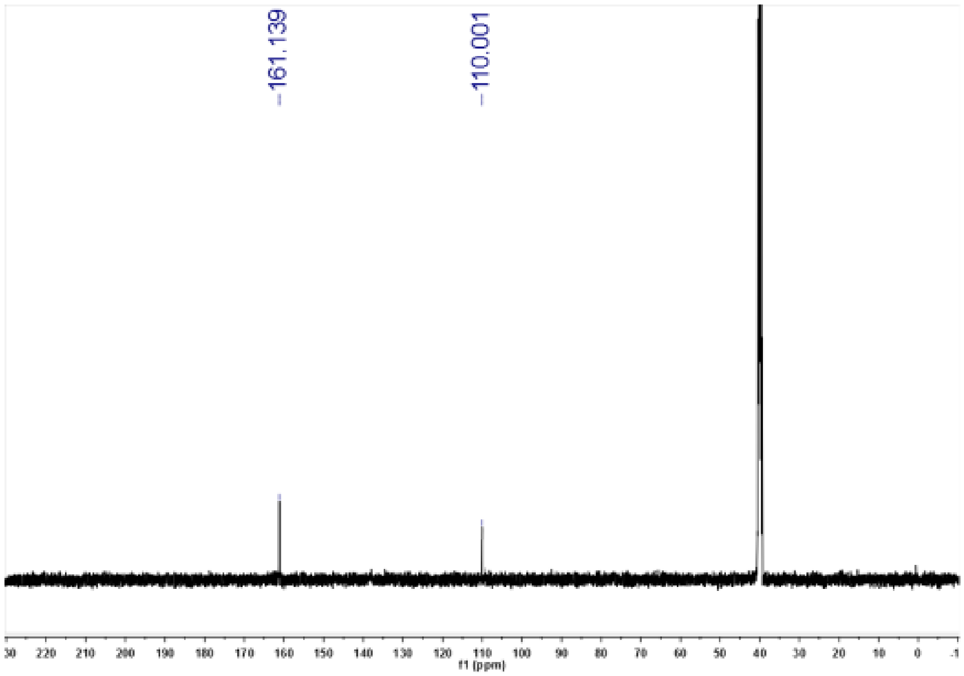

S3. Complete Crystallographic Data

Table S1. Atomic coordinates and equivalent isotropic displacement parameters of $3K_3BNOT\cdot 4H_2O$

Atom	Atom	Length/Å	Atom	Atom	Length/Å
K1	K6[1]	4.715(3)	K4	O17	2.744(3)
K1	K7[2]	4.529(10)	K4	O20A	3.046(10)
K1	K9[2]	4.584(5)	K4	O20B	2.98(3)
K1	O6	2.681(4)	K4	O21	2.765(3)
K1	O8	2.779(5)	K4	O25[5]	2.979(2)
K1	O18[2]	3.207(5)	K4	N16	3.301(3)
K1	O19[2]	2.833(6)	K4	N24[5]	3.152(3)
K1	O3[1]	3.053(5)	K5	O1[3]	3.029(3)
K1	O27[2]	2.520(10)	K5	O2[3]	2.926(3)
K1	N4[3]	3.221(4)	K5	O11[8]	2.768(3)
K1	N5[4]	3.241(11)	K5	O14A	3.191(13)
K1	N15[2]	3.439(5)	K5	O15[8]	2.745(3)
K2	K3	3.9752(11)	K5	O16	3.151(3)

Atom	Atom	Length/Å	Atom	Atom	Length/Å
K2	O26^{5}	2.761(2)	K5	O23	2.686(3)
K2	O9	2.723(3)	K5	O25	2.804(2)
K2	O17	2.691(3)	K5	N1^{3}	3.371(3)
K2	O21^{5}	2.756(3)	K5	N10^{3}	3.097(3)
K2	N3^{3}	2.822(3)	K6	O5^{1}	2.844(3)
K2	N12	3.106(3)	K6	O6^{1}	2.902(3)
K2	N19^{5}	3.346(3)	K6	O7^{9}	3.228(3)
K2	N20^{5}	3.267(3)	K6	O10	2.786(3)
K2	N21^{5}	3.178(3)	K6	O11	2.996(3)
K2	C10^{5}	3.177(3)	K6	O22^{7}	2.908(3)
K2	C11^{5}	3.276(3)	K6	O23^{7}	2.901(2)
K3	O26^{5}	2.767(2)	K6	O3	2.773(3)
K3	O2	2.707(3)	K6	N7^{1}	3.291(3)
K3	O3A	2.711(8)	K6	N8	3.252(3)
K3	O3B	2.871(10)	K6	N11^{1}	2.860(3)
K3	O9	3.019(4)	K6	N17^{7}	3.191(3)
K3	O12^{6}	2.892(2)	K7	O1^{1}	2.932(3)
K3	O13^{6}	2.879(2)	K7	O14A	2.715(9)
K3	O13	2.726(2)	K7	O14B	2.667(18)
K3	O25^{7}	3.345(2)	K7	O15	3.133(3)
K3	N9^{6}	3.305(3)	K7	O22	3.304(3)
K3	N10	3.021(3)	K7	O3^{3}	2.815(3)
K3	N11	3.466(3)	K7	O27	2.658(3)
K4	O26^{5}	2.804(2)	K7	N3^{1}	3.373(3)
K4	O2^{6}	2.812(3)	K7	N12^{8}	2.807(3)
K4	O13	2.722(2)	K7	N13	3.310(3)
K4	O16^{5}	2.767(3)	K8	O3A^{6}	3.074(10)
K4	O17^{5}	3.069(3)	K8	O3B^{6}	3.002(9)
K8	O4^{6}	3.322(3)	O19	N15	1.259(4)
K8	O7^{9}	2.973(3)	O20A	N16	1.261(7)
K8	O8^{9}	3.267(3)	O20B	N16	1.231(14)
K8	O10	2.737(3)	O21	N16	1.258(4)
K8	O12	2.613(2)	O22	N17	1.255(4)
K8	O18	2.694(3)	O23	N17	1.242(4)

Atom	Atom	Length/Å	Atom	Atom	Length/Å
K8	O20B	3.34(3)	O25	N24	1.247(3)
K8	O28	2.809(13)	O13	N9	1.276(3)
K8	O29	3.068(14)	N1	C1	1.387(4)
K8	N19[7]	3.018(3)	N2	C1	1.359(5)
K9	O4[10]	2.873(3)	N3	N4	1.345(4)
K9	O7[10]	2.807(3)	N3	C2	1.350(4)
K9	O19	2.674(3)	N4	N5	1.346(4)
K9	N3[10]	3.167(3)	N5	C3	1.348(4)
K9	N4[10]	3.072(3)	N6	C4	1.384(4)
K9	N5[10]	3.059(3)	N7	C4	1.374(4)
K9	N20	2.945(3)	N8	C5	1.386(4)
K9	N21[11]	2.883(3)	N9	C5	1.365(4)
K9	N21	2.896(3)	N10	N11	1.340(4)
K9	C2[10]	3.166(3)	N10	C6	1.353(4)
K9	C3[10]	3.105(3)	N11	N12	1.343(4)
K9	C10	3.208(3)	N12	C7	1.353(4)
O26	N24	1.273(4)	N13	C8	1.389(4)
O1	N1	1.244(4)	N14	C8	1.363(4)
O2	N1	1.252(4)	N15	C9	1.366(5)
O3A	N2	1.316(9)	N16	C9	1.395(5)
O3B	N2	1.250(8)	N17	C12	1.389(4)
O4	N2	1.265(4)	N19	N20	1.345(4)
O5	N7	1.268(4)	N19	C11	1.353(4)
O6	N7	1.245(4)	N20	N21	1.349(4)
O7	N6	1.258(4)	N21	C10	1.345(5)
O8	N6	1.231(4)	N24	C12	1.372(4)
O10	N8	1.250(4)	C1	C2	1.459(4)
O11	N8	1.253(4)	C2	C3	1.374(5)
O12	N9	1.242(3)	C3	C4	1.466(5)
O14A	N13	1.258(7)	C5	C6	1.460(4)
O14B	N13	1.265(13)	C6	C7	1.386(5)
O15	N13	1.244(4)	C7	C8	1.458(5)
O16	N14	1.253(4)	C9	C10	1.455(5)
O17	N14	1.270(4)	C10	C11	1.389(5)

Atom	Atom	Length/Å	Atom	Atom	Length/Å
O18	N15	1.254(4)	C11	C12	1.452(5)

Table S2. Detailed parameters of detonation performance calculations (EXPLO5 v6.05)

Parameter	Value
Chemical formula	$K_3C_4N_7O_8$
Molar mass (g·mol^{-1})	482.57
Crystal density (g·cm^{-3})	2.077
Heat of formation (kJ·mol^{-1})	-135.3
Oxygen balance (%)	-16.6
Detonation velocity (m·s^{-1})	8836
Detonation pressure (GPa)	28.6
Detonation temperature (K)	3215
Detonation products (mol·kg^{-1})	N_2: 7.25, CO_2: 2.07, K_2O: 3.11

Detonation parameters were calculated using the EXPLO5 v6.05 software with the built-in BKWC equation of state and standard explosive database. The input parameters were as follows:

Chemical formula: $K_3C_4N_7O_8$

Molar mass: 482.57 g·mol^{-1}

Crystal density: 2.077 g·cm^{-3} (193K)

Heat of formation: −135.3 kJ·mol^{-1} (calculated via Born-Haber cycle using the Kapustinskii equation for lattice energy estimation)

Oxygen balance: −16.6% (calculated for CO_2 and H_2O as oxidation products)

File S1. Crystallographic information file (CIF) for $3K_3BNOT{\cdot}4H_2O$ (CCDC No. 1491887)Note: The CIF file has been deposited in the Cambridge Crystallographic Data Centre and is available separately upon request.
